# Inverse Molecular Docking Elucidating the Anticarcinogenic Potential of the Hop Natural Product Xanthohumol and Its Metabolites

**DOI:** 10.3390/foods11091253

**Published:** 2022-04-26

**Authors:** Katarina Kores, Zala Kolenc, Veronika Furlan, Urban Bren

**Affiliations:** 1 Laboratory of Physical Chemistry and Chemical Thermodynamics, Faculty for Chemistry and Chemical Technology, University of Maribor, Smetanova 17, SI-2000 Maribor, Slovenia; katarina.kores@um.si (K.K.); zala.kolenc@um.si (Z.K.); veronika.furlan@um.si (V.F.); 2 Department of Applied Natural Sciences, Faculty of Mathematics, Natural Sciences and Information Technologies, University of Primorska, Glagoljaška 8, SI-6000 Koper, Slovenia

**Keywords:** xanthohumol, metabolites, inverse molecular docking, anticarcinogenic effects

## Abstract

Natural products from plants exert a promising potential to act as antioxidants, antimicrobials, anti-inflammatory, and anticarcinogenic agents. Xanthohumol, a natural compound from hops, is indeed known for its anticarcinogenic properties. Xanthohumol is converted into three metabolites: isoxanthohumol (non-enzymatically) as well as 8- and 6-prenylnaringenin (enzymatically). An inverse molecular docking approach was applied to xanthohumol and its three metabolites to discern their potential protein targets. The aim of our study was to disclose the potential protein targets of xanthohumol and its metabolites in order to expound on the potential anticarcinogenic mechanisms of xanthohumol based on the found target proteins. The investigated compounds were docked into the predicted binding sites of all human protein structures from the Protein Data Bank, and the best docking poses were examined. Top scoring human protein targets with successfully docked compounds were identified, and their experimental connection with the anticarcinogenic function or cancer was investigated. The obtained results were carefully checked against the existing experimental findings from the scientific literature as well as further validated using retrospective metrics. More than half of the human protein targets of xanthohumol with the highest docking scores have already been connected with the anticarcinogenic function, and four of them (including two important representatives of the matrix metalloproteinase family, MMP-2 and MMP-9) also have a known experimental correlation with xanthohumol. Another important protein target is acyl-protein thioesterase 2, to which xanthohumol, isoxanthohumol, and 6-prenylnaringenin were successfully docked with the lowest docking scores. Moreover, the results for the metabolites show that their most promising protein targets are connected with the anticarcinogenic function as well. We firmly believe that our study can help to elucidate the anticarcinogenic mechanisms of xanthohumol and its metabolites as after consumption, all four compounds can be simultaneously present in the organism.

## 1. Introduction

Natural compounds are known to possess antioxidative, antimicrobial, anti-inflammatory, and anticarcinogenic properties and are generally considered to be pharmacologically safe [1]. An ever increasing use of natural dietary supplements has been observed for the prevention and/or treatment various cancers [2,3]. Xanthohumol represents a prenylated chalcone and is mainly found in the female inflorescence of the hop (*Humulus lupulus* L.). As hops are most commonly used in brewing industries (for flavoring, bitterness, and preservation), beer represents the main dietary source of xanthohumol [4,5]. Xanthohumol can be isomerized into isoxanthohumol by a thermal reaction in the hot wort. Because of the hydrophobic character of xanthohumol, isoxanthohumol also demonstrates better solubility in beer [6]. Likewise, xanthohumol can be non-enzymatically isomerized into isoxanthohumol and enzymatically into 8-prenylnaringenin or 6-prenylnaringenin by the hepatic metabolism and microbiota enzymes, respectively [3,7].

It has already been reported that xanthohumol can inhibit the growth of breast, colon, hepatocellular, ovarian, pancreatic, and prostate cancer cells as well as leukemia cells [2,3]. Xanthohumol mostly inhibits cell proliferation and induces cell apoptosis (caspase-dependent and caspase-independent) [1,8]. Moreover, xanthohumol has been shown to inhibit tumor cell invasion and the nuclear factor ĸB (NF-ĸB), which is, in addition to the invasion, also associated with cell proliferation, angiogenesis, and the metagenesis of various cancer cells [1,2]. The precise molecular mechanisms through which xanthohumol exerts its anticarcinogenic properties, however, remain poorly understood [3].

**Figure 1:**
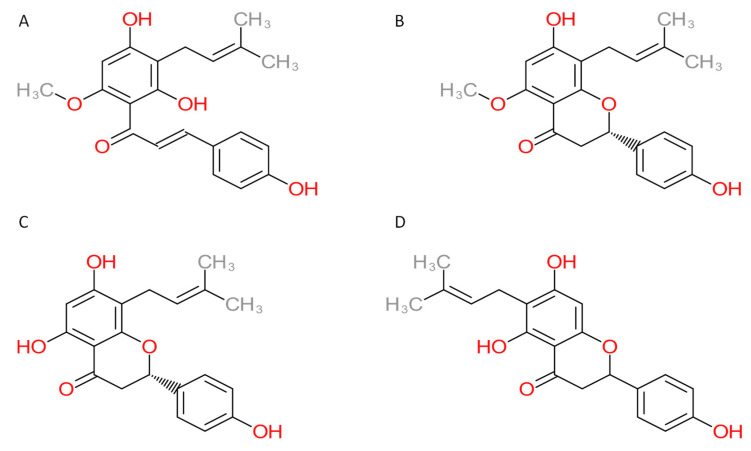
Chemical structures of compounds: (**A**) xanthohumol, (**B**) isoxanthohumol, (**C**) 8-prenylnaringenin, and (**D**) 6-prenylnaringenin.

Inverse molecular docking represents a novel, state-of-the-art approach used in drug discovery [9] and drug repurposing [10,11]. It has already been a useful tool for finding new potential targets of already established drugs [10]. Chen [12] used the approach to reveal the potential new targets of tanshinone IIA, used in the treatment of acute promyelocytic leukemia. New potential side effects of drugs can also be explored [13]. For example, a study by Kores et al. successfully proposed the new potential side effects of two drugs used to treat insulin resistance—troglitazone and rosiglitazone—and potential mechanisms of known side effects were proposed [14]. The approach has also been used to establish the molecular mechanisms of the health-promoting effects of natural compounds such as resveratrol, curcumin, and polyphenolic compounds from rosemary [15,16,17]. With the application of the inverse docking approach, a novel protocol named inverse docking fingerprinting was also developed, which has been used to find approved drugs with similar effects on protein targets from the *Coronaviridae* family [18]. In this study, we used the inverse molecular docking protocol to search for potential human protein targets of xanthohumol and its metabolites—isoxanthohumol 8-prenylnaringenin and 6-prenylnaringenin (Figure 1)—in order to determine their anticarcinogenic modes of action.

## 2. Materials and Methods

### 2.1. Inverse Molecular Docking

**Figure 2:**
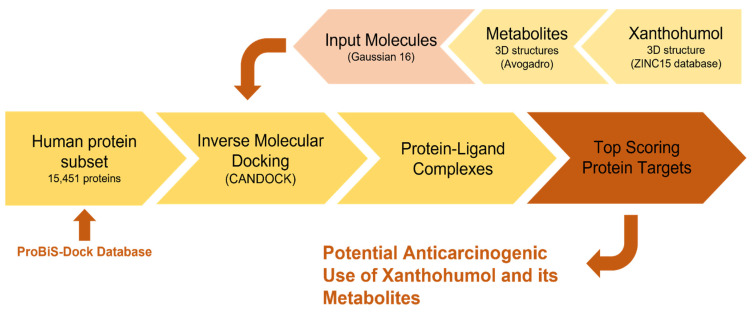
The workflow of the protein target prediction approach. Using inverse molecular docking, xanthohumol was docked into target human proteins from the Protein Data Bank (PDB) with predicted binding sites from the ProBiS-Dock database. Inverse molecular docking was performed with the CANDOCK algorithm.

An inverse molecular docking approach (Figure 2) was applied to 15,451 human protein structures from the Protein Data Bank (PDB). The binding sites for small molecules were identified based on the evolutionary conservation of the binding-site amino acids prepared for the inverse molecular docking. With the reduction of the docking space and by solely focusing on the protein binding sites, the time and complexity of the inverse molecular docking were also shortened. The preparation of the protein binding site (ProBiS-Dock) database was carried out in several steps. The binding site for a specific protein was determined with the use of the binding site similarity of all PDB entries with co-crystallized ligands of the same protein. The procedure is described in detail by Konc et al. and Štular et al. [19,20] and has been successfully used before to obtain mechanistic insights into the side effects of troglitazone and rosiglitazone [14] as well as to determine the potential applications of resveratrol and rosemary polyphenols [15,17]. Moreover, the protocol has been successfully applied in a novel drug repurposing approach named inverse docking fingerprinting [18].

The CANDOCK algorithm [21] applies a hierarchical approach for the reconstruction of small molecules from the atomic grid using generalized statistical potential functions and graph theory. The docking scores represent approximations of the relative binding free energies and have arbitrary units. The algorithm works by following the subsequent steps. First, it takes a small molecule and divides it into fragments. Then, these fragments are docked into protein binding sites from the ProBiS-Dock database using knowledge-based scoring methods. The best-docked poses of fragments are then selected, and a fast-maximum clique algorithm is applied [22] to link them together. In the process of ligand reconstruction, the iterative dynamics of the amino acid active site is used for its better placement in the binding pocket. In the last step, the minimization of the docking score is performed [14,20,21].

### 2.2. Preparation and Execution of Inverse Docking Procedure Using CANDOCK Algorithm

The inverse molecular docking of xanthohumol and its metabolites into the prepared human protein database was performed according to the following steps:

Step 1. Xanthohumol and its three metabolites—isoxanthohumol, 8-prenylnaringenin, and 6-prenylnaringenin—were used as ligands. The 3D structure of xanthohumol was obtained from the ZINC15 database [23], and the structures of the metabolites were prepared with the Avogadro software [24]. All molecules were optimized with Gaussian 16 [25] by applying the Hartree–Fock calculation and a flexible 6-31G basis set. Optimization was performed to correct bond lengths and angles, especially for metabolites prepared by hand.

Step 2. The ProBiS-Dock database was prepared, as described by Konc et al. [19].

Step 3. The prepared molecules and database were used as the inputs for the CANDOCK algorithm. The calculations were caried out on an in-house server.

Step 4. We took into consideration all the ligand poses for each target protein, and the one with the lowest docking score was considered the best. The protein–ligand complexes were obtained and ranked from these conformations, and a ranked list of protein targets was prepared. 

**Figure 3:**
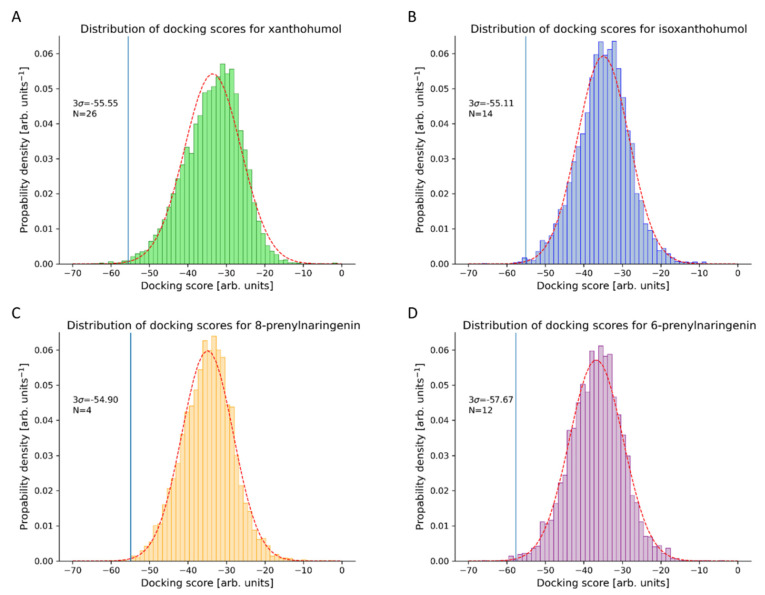
Normal distribution fitting graphs of inverse docking scores for xanthohumol, isoxanthohumol, 8-prenylnaringenin, and 6-prenylnaringenin. 3σ is the designation for 99.7% confidence interval, and the N represents the number of proteins that fit these preselected criteria.

Step 5. The best-ranking proteins from the list were selected based on the assumption of normal distribution and the 99.7% confidence criteria (Figure 3). Proteins with their docking scores below the selected threshold were considered as potential human protein targets of xanthohumol and its metabolites. 

Step 6. For these protein targets, the known connection with cancer was investigated. Numerous novel potential protein targets, into which the four investigated molecules were successfully docked, were identified. 

### 2.3. Method Validation Using Retrospective Metrics 

The validation was caried out using receiver operating characteristics (ROC) [26], enrichment [27], and predictiveness curves (PC) [28]. On the ROC metric plot, the correlation between the true (TPF, *y*-axis) and the false (FPF, *x*-axis) positive fraction is shown. In our case, the TPF represents the experimentally confirmed protein targets of xanthohumol from the ChEMBL database [29] with the corresponding PDB entry, while the FPF shows protein targets where xanthohumol was successfully docked. Each point of the ROC curve therefore represents a unique FPF/TPF pair, which allows for the estimation of the overall success of a screening method in the discrimination of experimentally confirmed protein targets from protein targets that have not yet been experimentally confirmed. The area under the ROC (ROC AUC) is used as a measure of the overall discrimination of the experimentally confirmed protein targets. ROC AUC values higher than 0.5 signify that the protocol is effective in distinguishing the experimentally confirmed proteins from the unconfirmed protein targets [26]. Enrichment curves [27] and PC [28] allow the quantification of the early detection of experimentally confirmed protein targets by visualizing the TPF (*y*-axis) versus the entire data set on a logarithmic scale (*x*-axis). By applying the PC, a score threshold for potential protein targets to be tested experimentally can then be determined [28]. The early detection quantification is determined with an enrichment factor of 1% for screened compounds (EF1%) [30], the Boltzmann-enhanced discrimination of ROC (BEDROC) [27], and the robust initial enhancement (RIE) [30]. Therefore, EF1%, BEDROC, and RIE are used to quantify the early recognition of experimentally confirmed protein targets.

The standardized total gain (TG) is also calculated using the PC and represents the discrimination of experimentally confirmed proteins imputable to the variation of the docking scores over the entire data set. ROC AUC values over 0.5 combined with TG over 0.25 signify that docking score variations are relevant in the discrimination of experimentally confirmed protein targets [28]. To cover all aspects of the presented analysis, the web-based interactive application, Screening Explorer [31], was applied. The validation of the algorithm using retrospective metrics and redocking procedures has also been extensively performed in previous studies [14,15,16,17,21].

## 3. Results and Discussion

### 3.1. Novel Human Protein Targets of Xanthohumol

The calculated conformations of xanthohumol, docked in human protein binding sites, were arranged according to their docking scores. The docking scores were assumed to be normally distributed, and by using the 99.7% confidence interval, we were able to identify 26 top-scoring xanthohumol protein targets, with a docking score cut-off of −55.56 arbitrary units. The predicted docking scores, protein functions, and reported experimental correlation of identified proteins with anticarcinogenic functions are collected in Table 1.

Out of 26 identified potential protein targets of xanthohumol (by using a threshold of 99.7%), 16 are known to have a connection with cancer. From these, four proteins also have an experimental correlation with xanthohumol. Based on these results, the identified proteins provide a promising opportunity to create a novel design process for chemopreventive compounds. In the following subsections, we will describe in detail the protein targets of xanthohumol that are, to the best of our knowledge, connected to cancer.

We identified the N-lysine methyltransferase SMYD2, whose function is to suppress cell proliferation and directly regulate p53 function [33]. The tumor suppressor p53 represents one of only a few non-histone proteins known to be regulated by lysine methylation. The methylation of p53 by SMYD2 can result in a repression of its function, which makes the SMYD2 a potential tumor suppressor. [32]

Acyl-protein thioesterases (APT) are members of a protein group involved in depalmitoylation processes. The palmitoylation/depalmitoylation cycle takes place by moving proteins between the plasma membrane and the Golgi apparatus. This dynamic cycle is tightly regulated by palmitoyl transferases (palmitoylation) and acyl protein thioesterases 1 and 2 (APT-1, APT-2) (depalmitoylation). Human APT-1 and APT-2 represent major components in the control of the palmitoylation dynamic cycle of Ras oncogenes [34]. For the NRAS and HRAS proteins, the described cycle was identified for the first time in the study of Baekkeskov et al. [66]. A recent report described how the disruption of the HRAS and NRAS palmitoylation/depalmitoylation cycle by the inhibition of APT-1 and APT-2 resulted in reduced growth and signaling in cells with oncogenic HRAS/NRAS mutations [34].

Another protein identified was glutamate carboxypeptidase III (GCPIII), a metalloenzyme that belongs to the transferrin receptor⁄glutamate carboxypeptidase II (GCPII) superfamily [35]. GCPIII has an evolutionary connection with GCPII (67% sequence similarity). Human glutamate carboxypeptidase II (GCPII) cleaves N-acetyl-L-aspartyl-glutamat in the brain, liberating free glutamate. The inhibition of GCPII has been shown to be neuroprotective in models for stroke and other neurodegenerations. In prostate cancer, it is also known as a prostate-specific membrane antigen and a prostate cancer marker [36].

Protein arginine methyltransferase 6 (PRMT6) is a nuclear enzyme whose function is to methylate arginine residues on histones and transcription factors. Similar to other PRMTs, PRMT6 is overexpressed in several cancer types [37,38,39]. Due to its function, PRMT6 is considered as a potential anti-cancer drug target [37]. Therefore, the dysregulation of PRMTs is usually associated with diverse types of cancer [38]. A recent study showed that PRMT6 represents a promising target for pharmaceutical drug development based on the molecular mechanism underlying PRMT6-mediated colorectal cancer cell apoptosis, which should be investigated further [38].

The main function of identified matrix metalloproteinase-9 (MMP-9) is its proteolytic activity in the extracellular environment. MMP-9 plays a role in basement membrane degradation since basement membrane contains collagens. During tumor development, basement membrane destruction is usually an essential step that supports tumor invasion and metastases [40,41]. Carcinogenesis includes several important processes such as migration, invasion, metastasis, and angiogenesis, and all these processes are closely related to the extracellular environment. MMP-9 plays an important role in extracellular environment remodeling and membrane protein cleavage, and it is found to be widely associated with cancer pathologies. MMP-9 is additionally recognized as a cancer biomarker due to its function in the promotion of cancer development. Further investigations are focused on MMP-9 inhibitors [40]. Xanthohumol was reported as one of the MMP-9 and MMP-2 inhibitors. In addition, MMP-9 may represent a valuable anti-angiogenic agent in the treatment of chronic diseases such as cancer and inflammation [41].

The mitogen-activated protein kinase (MAPK) pathway controls the growth and survival of a broad spectrum of human tumors [42]. As a consequence of the abnormal activation of the MAPK pathway, we see an uncontrolled cell proliferation [67]. Several inhibitors of MAPK signaling have therefore been developed. Inhibiting this pathway in animal models has resulted in no apparent abnormalities. However, MAPKs do have a twofold reduction in the number of mature thymocytes [42]. A direct impact of xanthohumol on MAPK has not yet been published, but on the other hand, xanthohumol treatment triggered the MAPK (isoform p38) and inhibited the paraptosis of HL-60 leukemia cells. It has also been demonstrated for the first time that xanthohumol treatment can induce the paraptosis of leukemia cells through the activation of p38 MAPK signaling [43].

Poly (ADP-ribose) polymerase (PARP) represents a family of proteins involved in several cellular processes such as DNA repair, genomic stability, and programmed cell death. PARP is an enzyme included in the post-translational modifications of proteins as a response to numerous endogenous and environmental genotoxic agents [44]. The direct connection of PARP2 with anticarcinogenic functions was not revealed; this was only found to be true for PARP. The research paper of Donawho et al. [45] revealed a potential PARP inhibitor (ABT-888), which passed a lot of tests and was proposed for clinical evaluation as an anticancer agent. Xanthohumol (dependent on dosage) inhibited the proliferation of 40–16 colon carcinoma cells (in vitro tests). Besides the inhibition of cell growth, the cytotoxic effects of xanthohumol were also observed. Cell death caused by xanthohumol was mediated by the induction of apoptosis. This is an indication of PARP cleavage, of the involvement of the death receptor, and of the mitochondrial pathway via the activation of caspases −3, −7, −8, and −9 as well as via the modulation of the Bcl-2 protein expression [46]. Other published research works have shown that the induction of apoptosis increases when applying xanthohumol in combination with honokiol because of their synergistic effects [47].

The family of RAB6 proteins consists of three different isoforms: RAB6A, RAB6A, and RAB6B. RAB6B is predominantly expressed in the brain. Wanschers et al. [48] concluded from their data that the brain-specific RAB6B is linked to the dynein/dynacitin complex. The authors suggested the regulatory role of RAB6B in the retrograde transport of cargo in neutral cells. Only the Ras-related protein Rab-6B and lysozyme C were identified as CEA (carcinoembryonic antigen)-interacting proteins. The CEA-mediated radiation response appears to vary due to the characteristics of the individual cancer cells. The lysozyme C and Rab subfamily proteins should therefore play a role in the link between CEA and tumor response to radiation [49]. The overactivity of Ras signaling can also lead to cancer. The three Ras genes in humans (HRas, KRas, and NRas) are the most common oncogenes in human cancer; mutations that permanently activate Ras are found in human carcinogenesis. For this reason, Ras inhibitors are being studied as a potential treatment for cancer and other diseases with the Ras overexpression [49].

The SET and MYND domain containing protein 3 (SMYD3) represents a novel histone lysine methyltransferase. The main function of SMYD3 is the regulation of chromatin during the development of myocardial and skeletal muscles. Lysine methylation plays a vital role in histone modification. The consequences of the downregulation of lysine methyltransferases have been observed in multiple human cancer types (esophageal squamous cell carcinoma, gastric cancer, hepatocellular carcinoma, cholangiocarcinoma, breast cancer, prostate cancer, and leukemia). SMYD3 also plays a crucial role in the transcriptional regulation of carcinogenesis and the development of human cancers. Moreover, the SMYD3 has been suggested as a potential prognostic marker [50].

Matrix metalloproteinase (MMP)-14 represents a membrane-bound MMP that plays a critical role in conferring cells with the ability to remodel and penetrate the extracellular matrix [51]. High MMP-14 expression is associated with the early death of patients with breast cancer and is correlated with lymph node metastases, progression, invasion, poor clinical stage, larger tumor size, and an increased tumor stage. The inhibition of MMPs has been extensively used as one of the therapeutic strategies in cancer treatment, usually with compounds containing zinc-chelating groups [51].

Ribosomal protein S6 kinase alpha 5 (RPS6KA5) possesses several biochemical functions, for example, ATP binding, histone kinase activity (H3-S10 specific), and magnesium ion binding. RPS6KA5 siRNAs were co-delivered with anti-apoptotic Mcl-1 siRNAs, and RPS6KA5 was found to be one of the appropriate candidates for simultaneous silencing with Mcl-1. In both wild type and resistant xenografts in nude mice, the double silencing of Mcl-1/RPS6KA5 also led to an improved inhibition of tumor growth by exhaustion from chemotherapy [52].

Aldo-keto reductase family 1, member C1 (AKR1C1) is involved in maintaining steroid hormone homeostasis, prostaglandin metabolism, and the metabolic activation of polycyclic aromatic hydrocarbons [54]. Microarray analysis revealed AKR1C1 to be up-regulated in metastatic lesions, which has been verified in metastatic human bladder cancer specimens. Decreased invasion caused by AKR1C1 knockdown suggests a novel role for AKR1C1 in cancer invasion. AKR1C1 is also recognized to be a potent molecular target in invasive bladder cancer treatment [54].

It is probable that dimethyladenosine transferase (DIMT) is involved in the pre-rRNA procedure, which leads to the production of the small subunit rRNA. The direct connection of DIMT with cancer diseases has not been reported yet, but a recent article about DIMT1 shows that it is closely associated with the proliferation, apoptosis, invasion, and migration to tumor cells. Liu et al. [56] revealed that DIMT1 represents a useful molecular biomarker for predicting the progression and prognosis of patients with gastric carcinoma based on the overexpression of its gene. On the basis of its high expression, we assume that its inhibition could help in defeating cancer [56].

Gelatinase A (together with gelatinase B) is a member of the gelatin-binding structure group and is a part of the matrix metalloproteinases (MMPs). Gelatinase A is therefore also known as MMP-2 and gelatinase B as MMP-9. These two gelatinases play an important role in the development and progression of colorectal cancer [59]. Gelatinases A and B have been associated with the invasive and metastatic behavior of malignant tumors. Cancer cells and stroma represent the basic units of tumors. The cancer cells could be the source of gelatinases in certain cases, but frequently, only stromal cells express gelatinases. Invasion and metastasis are only connected to the active forms of MMP-2 and MMP-9, while the mRNA expression or protein level is not considered a criterion for determining MMP-2/MMP-9 association with cancer [59]. To the best of our knowledge, the direct effect of xanthohumol on gelatinase A has not yet been published. But 2-hydroxychalcone and xanthohumol manifest inhibitory effects on the proliferation, MMP-9 expression, and invasive phenotype of the methotrexate-resistant cells, MDA-MB-231. These results suggest the potential application of chalcones (similar to xanthohumol) as anticancer agents, which can alleviate the malignant progression of triple negative breast cancers [60].

Dihydrofolate reductase (DHFR) converts dihydrofolate into tetrahydrofolate and plays a crucial role in cell metabolism and cellular growth [61]. Kalogris et al. [68] identified the compound sanguinarine as a potential inhibitor of DHFR due to its ability to impair its enzymatic activity, even in the methotrexate-resistant MDA-MB-231 cells. Considering these findings, sanguinarine represents a promising anticancer drug for the treatment of breast cancer. There are already certain drug substances (such as methotrexate) in use which act as DHFR inhibitors in the treatment of leukemia osteosarcoma, breast cancer, or head and neck cancer [62]. Therefore, we hypothesize that xanthohumol as a DHFR inhibitor may have similar results.

Peroxisome proliferator-activated receptor delta (PPARδ) is involved in differentiation, lipid accumulation, directional sensing, polarization, and migration in keratinocytes. Gupta et al. [64] concluded that PPARδ is aberrantly expressed in colorectal tumors, and that endogenous PPARδ is transcriptionally responsive to PGI_2_. Barak et al. [63] also revealed PPARδ as a protein with an oncogenic function and as a potent target for the suppression of colorectal tumors.

**Figure 4:**
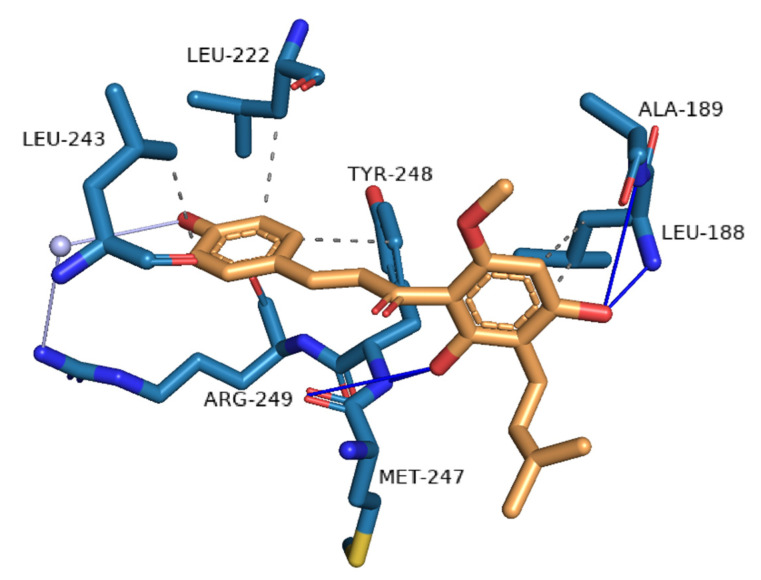
Analysis of interactions between the binding site of matrix metalloproteinase 9 (blue) and the docked xanthohumol (orange). Hydrogen bonds are depicted with blue lines, water bridges with light purple, and hydrophobic interactions with gray.

A detailed analysis of the interactions of the docked xanthohumol within the binding pocket of matrix metalloproteinase 9 (PDB ID 4jij) was also performed (Figure 4) to reveal the structure of the bound complex for the first time. In the binding site, xanthohumol formed hydrogen bonds with four amino acids, Leu-188, Ala-189, Met-247, and Tyr-248. A water bridge was also formed with Arg-149 through the conserved water molecule in the binding site. Moreover, hydrophobic interactions were formed with amino acids Leu-188, Leu-222, Leu-243, and Tyr-248. The strong hydrogen bonds and semi-strong hydrophobic interactions are consistent with the high docking score.

### 3.2. Novel Human Protein Targets of Isoxanthohumol, 8-Prenylnaringenin, and 6-Prenylnaringenin

The calculated conformations of isoxanthohumol, 8-prenylnaringenin, and 6-prenylnaringenin, docked into human protein binding sites, were ordered according to their docking scores. The docking scores were assumed to be normally distributed (Figure 3), with the 99.7% confidence interval <−55.11 arb. units for isoxanthohumol, <−54.90 arb. units for 8-prenylnaringenin, and <−57.67 arb. units for 6-prenylnaringenin. By applying the threshold at 99.7%, we have indeed focused on the top-scoring targets of all three xanthohumol metabolites. The identified proteins with predicted docking scores, protein functions, and reported experimental correlations with anticarcinogenic functions are described in the Supplementary Material, Tables S1–S3. We identified 14 proteins with docked isoxanthohumol, 4 proteins with docked 8-prenylnaringenin, and 12 proteins with docked 6-prenylnaringenin. For the proteins to which the xanthohumol metabolites were successfully docked, no experimental correlations with specific ligands can, to the best of our knowledge, be found. The last column in Tables S1–S3 specifies other xanthohumol metabolites docked to the same protein and their corresponding docking scores.

A total of 22 novel protein targets of isoxanthohumol, 8-prenylnaringenin, and 6-prenylnaringenin have known connections to cancer. From these, we would like to stress those proteins that appear to be some of the best targets for two or more of the investigated xanthohumol metabolites. Xanthohumol, isoxanthohumol, and 6-prenylnaringenin were successfully docked to acyl-protein thioesterase 2. Isoxanthohumol and 6-prenylnaringenin were successfully docked to folate receptor beta. Isoxanthohumol and 8-prenylnaringenin were successfully docked to dihydrofolate reductase. Xanthohumol was also successfully docked to the dihydrofolate reductase (PDB ID: 3gz9A), with the proteins with the PDB codes 3eigA and 3gz9A having a sequence similarity of 98.39%. The isoxanthohumol and 6-prenylnaringenin were successfully docked to 72 kDa type IV collagenase. Finally, xanthohumol and isoxanthohumol were successfully docked to Ras-related protein Rab-7. Based on these results, the identified proteins, where more than a single xanthohumol was docked, provide the most promising opportunity for the development of novel chemopreventive compounds.

**Figure 5:**
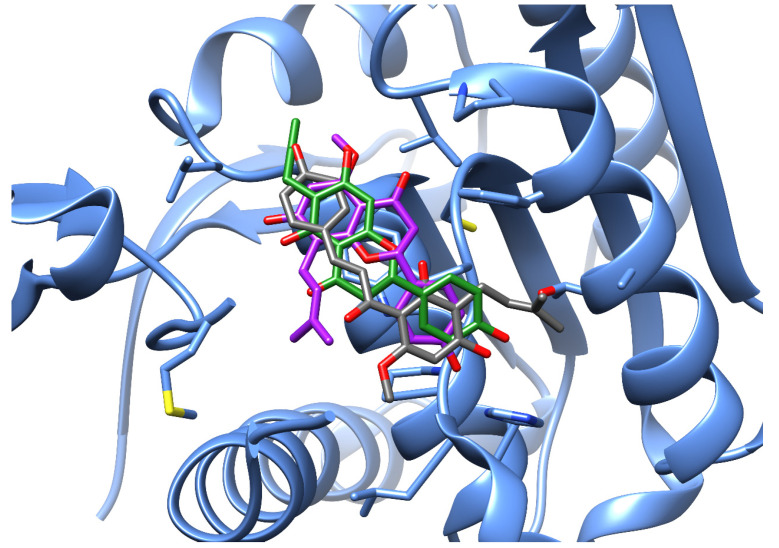
Comparison of the docked xanthohumol (gray), isoxanthohumol (purple), and 6-prenylnaringenin (green) to the acyl-protein thioesterase 2 (PDB ID 5syn). All docked molecules lie within the same binding pocket. The protein is depicted in a blue cartoon model.

**Figure 6:**
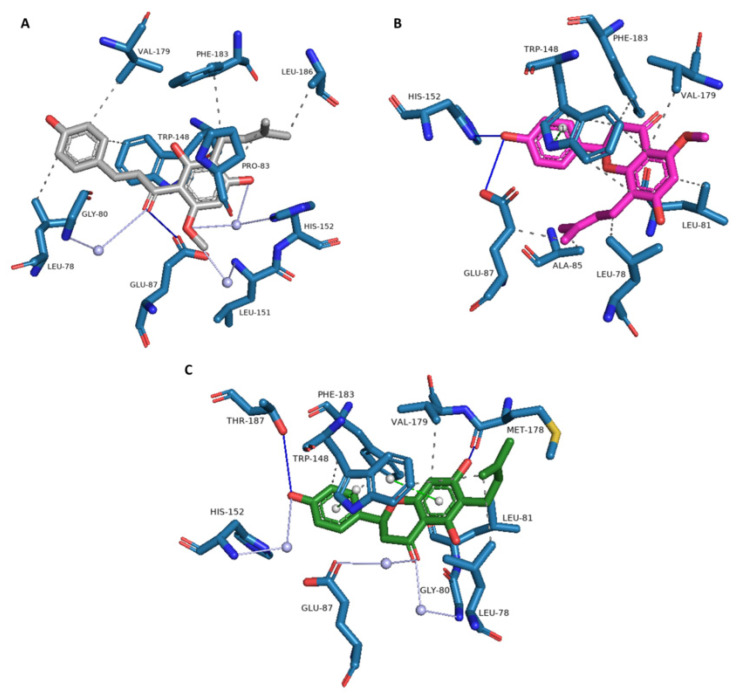
Analysis of interactions between the binding site of acyl-protein thioesterase 2 (blue) and (**A**) xanthohumol (gray), (**B**) isoxanthohumol (purple), and (**C**) 6-prenylnaringenin (green). Hydrogen bonds are represented by blue lines, water bridges by light purple, π-stacking by green, and hydrophobic interactions by gray.

A detailed analysis of the interactions of xanthohumol and two of its metabolites (isoxanthohumol and 6-prenylnaringenin) within the same binding pocket of acyl-protein thioesterase 2 was also performed (Figure 5). Their binding modes are detailed in Figure 6. The analysis showed that xanthohumol and its metabolites can form hydrogen bonds, water bridges, and π-stacking interactions. The presence of hydrophobic amino acids facilitates the formation of hydrophobic interactions as well. In the binding site of acyl-protein thioesterase 2, hydrophobic interactions were the most abundant, and four amino acids, Leu-78, Trp-148, Val-179, and Phe-183, were common to all three ligands. Strong hydrogen bonds were also formed with the amino acids Glu-87, His-152, Met-178, and Thr-187. Moreover, xanthohumol and 6-prenylnaringenin formed water bridges with the Gly-80, Glu-87, Leu-151, and His-152 amino acids through the conserved water molecules in the binding site. Furthermore, in the case of isoxanthohumol and 6-prenylnaringenin, π-stacking was also found with Trp-148. Strong hydrogen bonds and semi-strong hydrophobic interactions and water bridges are consistent with the high docking scores.

### 3.3. Method Validation

**Figure 7:**
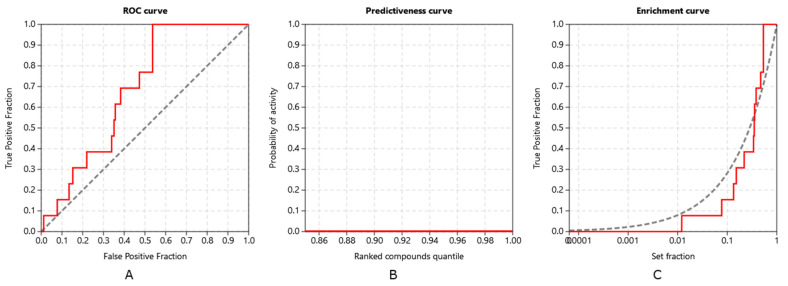
Validation of the inverse molecular docking protocol of xanthohumol against all human protein targets from the Protein Date Bank (PDB): (**A**) the receiver operating characteristics (ROC) curve; (**B**) the predictiveness curve; and (**C**) the enrichment curve.

Inverse molecular docking was performed on the set of 15,451 human proteins from the Protein Data Bank (PDB), from which 13 were experimentally confirmed targets of xanthohumol, and whose measured IC_50_ values for the xanthohumol binding were <10 µM. Our protocol successfully identified 10 of these confirmed target proteins. The ability of the CANDOCK algorithm to distinguish the confirmed protein targets of xanthohumol was assessed using the established retrospective metrics, receiver operating characteristics (ROC), predictiveness, and enrichment curves, shown in Figure 7. The validation was performed solely on xanthohumol, as there is not enough experimental data on its metabolites.

The validation using ROC, predictiveness, and enrichment curves shows that our protocol was indeed successful in discriminating between true protein targets of xanthohumol, with the area under the ROC (ROC AUC) at 0.684. Moreover, the early detections of targets, with the Boltzmann-enhanced discrimination of ROC (BEDROC) at 0.088, the robust initial enhancement (RIE) at 1.738, and the enrichment factor of 1% of screened compounds (EF1%) at 11.885, were satisfactory. The detection of true target proteins (TG of 0.210) in combination with the ROC AUC above 0.6 shows that the applied protocol is in good agreement with the experiments [28].

## 4. Conclusions

In this study, xanthohumol and its metabolites—isoxanthohumol, 8-prenylnaringenin, and 6-prenylnaringenin—were docked into human protein structures collected from the Protein Data Bank (PDB) [69]. The CANDOCK inverse docking algorithm [21] was used.

The most influential results of our study are the proteins, to which more than one investigated polyphenol was successfully docked with the lowest docking score value. Based on these, the acyl-protein thioesterase 2 should be exposed. Xanthohumol, isoxanthohumol, and 6-prenylnaringenin were successfully docked to acyl-protein thioesterase 2. Furthermore, the experimental correlation with the anticarcinogenic function of this protein was also found. Regarding the human protein targets to which xanthohumol was successfully docked in our study, more than half of them also have known experimental correlations with xanthohumol and anticarcinogenic function. Based on these results, the mechanisms of action could be substantiated, through which xanthohumol or its metabolites perform anticarcinogenic/chemoprotective activities.

On the other hand, the results obtained in this study (especially the proteins, to which more than a single compound was successfully docked) can be a promising source in the process of developing new chemopreventive compounds.

## Figures and Tables

**Table 1:** Potential human protein targets of xanthohumol.

PDB ID with Chain	Protein Name	Predicted Docking Score (arb. Units) *	Protein Function	Anticarcinogenic Function **	Correlation with Xanthohumol ***
5kjkA	N-lysine methyltransferase SMYD2	−64.79	Suppresses cell proliferation and directly regulates p53 function [32,33].	Yes [32]	No
5synA	Acyl-protein thioesterase 2	−64.13	Involved in depalmitoylation [34].	Yes [34]	No
3fedA	Glutamate carboxypeptidase III	−62.50	Involved in a variety of neuropathologies and malignancies such as glutamatergic neurotoxicity and prostate cancer [35].	Yes [36]	No
4y30A	Arginine N-methyltransferase 6	−62.00	Involved in the regulation of transcription process, signal transduction, human immunodeficiency virus pathogenesis, DNA damage response, and cell cycle progression [37,38,39].	Yes [38]	No
4jijA	Matrix metalloproteinase 9	−59.85	The main function of MMP-9 is proteolytic activity in the extracellular environment [40,41].	Yes [40]	Yes [41]
3e7oA	Mitogen-activated protein kinase 9	−59.84	The mitogen-activated protein kinase pathway controls the growth and survival of a broad spectrum of human tumors [42].	Yes [42]	Yes [43]
4zzxA	Poly [ADP-ribose] polymerase 2	−59.83	Involved in a number of cellular processes such as DNA repair, genomic stability, programmed cell death [44].	Yes [45]	Yes [46,47]
2ffqA	Ras-related protein Rab-6B	−59.78	Protein has a regulatory role in the retrograde transport of cargo in neutral cells [48,49].	Yes [49]	No
4lhwA	Ras-related protein Rab-8A	−59.77	Overactivity of Ras signaling can lead to cancer, and it was found in human tumors [49].	No	No
3ru0A	SET and MYND domain-containing protein 3	−59.53	Regulates chromatin during the development of myocardial and skeletal muscles [50].	Yes [50]	No
3ma2D	Matrix metalloproteinase-14	−59.24	Plays a critical role in conferring cells with the ability to remodel and penetrate the extracellular matrix [51].	Yes [51]	No
3lawA	Ras-related protein Rab-7a	−58.93	Ras inhibitors have been studied as a treatment for cancer and other diseases with Ras overexpression [49].	No	No
1zd9A	ADP-ribosylation factor-like 10B	−57.56	Physiological function of this protein is not known.	No	No
1vzoA	Ribosomal protein S6 kinase alpha 5	−57.32	Involved in several pathways such as MAPK signaling pathway, adrenergic signaling in cardiomyocytes, TNF signaling pathway, and possesses several biochemical functions such as ATP binding, histone kinase activity (H3-S10 specific), magnesium ion binding [52].	Yes [52]	No
5fbeA	Complement factor D	−57.27	The complement system plays an important role in the innate defense against common invading pathogens [53].	No	No
1mrqA	Aldo-keto reductase family 1 member C1	−56.90	Involved in maintaining steroid hormone homeostasis, prostaglandin metabolism, and metabolic activation of polycyclic aromatic hydrocarbons [54].	Yes [54]	No
2c73A	Amine oxidase (flavin-containing) B	−56.87	Plays an important role in neuroactive, vasoactive amines and is correlated with the production of neurotoxins in Parkinson’s disease [55].	No	No
1zq9A	Probable dimethyladenosine transferase	−56.86	Protein is involved in the pre-rRNA procedure, which leads to small-subunit rRNA production [56].	Yes [56]	No
5fa6A	NADPH- -cytochrome P450 reductase	−56.81	Protein is the redox partner of various P450s involved in primary and secondary metabolism [57].	No	No
2h44A	cGMP-specific 3′,5′-cyclic phosphodiesterase	−56.65	The protein catalyzes the hydrolysis of 3′,5′-cyclic nucleotides to their respective nucleoside 5′-monophosphates [58].	No	No
1ck7A	Gelatinase A	−56.53	Protein is a member of the gelatin-binding structure group and forms part of the matrix metalloproteinases (MMPs) [59].	Yes [59]	Yes [60]
1t91A	Ras-related protein Rab-7	−56.21	Ras signaling proteins have been found in human tumors [49].	No	No
3ghvA	Dihydrofolate reductase	−56.01	Converts dihydrofolate into tetrahydrofolate and plays a crucial role in cell metabolism and cellular growth [61].	Yes [62]	No
3gz9A	Peroxisome proliferator-activated receptor delta	−55.95	Protein is involved in differentiation, lipid accumulation, directional sensing, polarization, and migration of keratinocytes [63,64].	Yes [64]	No
2bzgA	Thiopurine S-methyltransferase	−55.80	Protein is an enzyme in the cytoplasm that is involved in catalyzing the S-methylation of thiopurine drugs [65].	No	No
1zc3A	Ras-related protein Ral-A	−55.56	Because of presence of Ras proteins in tumor, the Ras inhibitors have been studied [49].	No	No

* Knowledge-based docking scores with arbitrary units represent relative binding free energies of xanthohumol with a given protein. ** Reported experimental connection with anticarcinogenic function. *** Reported experimental correlation with xanthohumol.

## Data Availability

All data generated or analyzed during this study are included in the published article.

## Supplementary Materials

The following supporting information can be downloaded at: https://www.mdpi.com/article/10.3390/foods11091253/s1, Table S1: Potential human protein targets of isoxanthohumol, References [34,42,49,61,62,70,71,72,73,74,75,76,77,78] are cited in the Table S1, Table S2: Potential human protein targets of 8-prenylnaringenin, References [61,62,71,79,80] are cited in the Table S2, Table S3: Potential human protein targets of 6-prenylnaringenin, References [75,81,82,83,84,85,86,87,88,89,90,91] are cited in the Table S3. The predicted docking scores, protein functions, and reported experimental correlations with anticarcinogenic functions for isoxanthohumol, 8-prenylnaringenin, and 6-prenylnaringenin, respectively, are presented in Tables S1–S3.
