# Supplementary material for: Inverse Molecular Docking Elucidating the Anticarcinogenic Potential of the Hop Natural Product Xanthohumol and Its Metabolites
Source: Foods. 2022 Apr 26;11(9):1253. doi: 10.3390/foods11091253 (PMC9104229; doi:10.3390/foods11091253)
Supplement: Supplementary file 1 [file foods-11-01253-s001.zip › foods-1680759-supplementary.pdf]

Supplementary Material: Inverse Molecular Docking Elucidating the Anticarcinogenic Potential of the Hop Natural Product Xanthohumol and its Metabolites

Katarina Kores, Zala Kolenc, Veronika Furlan, and Urban Bren

Table S1. Potential Human Protein Targets of isoxanthohumol.

PDB ID with Chain	Protein Name	Predicted Docking Score [arb. units]*	Protein function	Anticarcinogenic Function**	Other Docked Ligands*** (docking score)
5synA	Acyl-protein thioesterase 2	-65.36	Protein is involved in depalmitoylation. [1]	Yes [1]	XH (-64.13), 6P (-58.59)
5aztA	Peroxisome proliferator-activated receptor alpha	-57.28	Peroxisome proliferator – activated receptors (PPAR) play an essential role in regulating the cancer cell growth. Activity of PPARα leads to the increase of oxidative stress and cell proliferation is induced while inhibiting the apoptosis. [2]	Yes [2]	-
4kmy A	Folate receptor beta	-57.21	Folate receptor beta is a member of the folate receptor family. Folate receptor beta is in conjunction with folate receptor alfa attached to cell membrane by glycosylphosphatidylinositol anchors. Both receptors are overexpressed in tumor cells and in tumor-associated macrophages. [3]	Yes [3]	8P (-57.38)
3dyn A	High affinity cGMP-specific 3',5'-cyclic phosphodiesterase 9A	-56.70	Protein regulates cGMP signalling independent of the nitric oxide pathway, and is considered as stress-induced heart disease potential therapeutic target. [4]	No	-

1i3kA	UDP-glucose 4-epimerase	-56.23	The UDP-glucose 4-epimerase is a member of the glycosyltransferase family. Its main function is protein and lipid glycosylation in normal and neoplastic cells. The activity of UDP-glucose 4-epimerase plays a significant role in the cell recognition, signalling, progression and metastasis of cancer cells. [5]	Yes [5]	-
2wef A	3'(2'), 5'-bisphosphate nucleotidase 1	-56.03	Physiological function of this protein is not well known to the best of our knowledge.	No	-
3f3yA	Bile salt sulfotransferase	-55.80	Bile salt sulfotransferase is a member of the sulfotransferase superfamily, which catalyze the sulfation of a multitude of xenobiotics, hormones and neurotransmitters. [6]	No	-
3eigA	Dihydrofolate reductase	-55.73	Converts dihydrofolate into tetrahydrofolate and plays a crucial role in the cell metabolism and cellular growth. [7]	Yes [8]	-
1qibA	72 kDa type IV collagenase	-55.59	The activity of 72kDa type IV collagenase may occur on many levels, such as transcriptional mechanisms, or extracellular activation of latent enzymes. The invasion of human tumor cells through basement membranes may be the result of the net type IV collagenolytic activity. [9]	Yes [9]	6P (-60.12)
2z5fA	Sulfotransferase family cytosolic 1B member 1	-55.48	Sulfotransferase family cytosolic 1B member 1 is a part of the sulfotransferase superfamily, which catalyze the sulfation of a multitude of xenobiotics, hormones and neurotransmitters. [6]	No	-
4qtbA	Mitogen-activated protein kinase 3	-55.36	The mitogen-activated protein kinase pathway controls the growth and survival of a broad spectrum of human tumors. [10]	Yes [11]	-

3vg9 A	Adenosine receptor A2a	-55.34	Adenosine receptor A2a is coupled (along with adenosine receptors A1, A2b and A3) to G-protein and to their complex, the released extracellular adenosine is binding. The extracellular adenosine complex, generated by this pathway inhibits the antitumor T cells and promotes the T cell apoptosis, what leads to the immune escape by the tumor. [12]	Yes [12]	-
5jgaA	TAK1 kinase - TAB1 chimera fusion protein	-55.21	The crystal structure of full length TAK1-TABs complex has been determined only recently. But the further structural insight into the protein and its activation properties have not been provided yet. [13]	No	-
1t91A	Ras-related protein Rab-7	-55.13	Ras signalling proteins have been found in human tumors. [14]	No	XH (-56.21)

* Knowledge-based docking scores with arbitrary units represent relative binding free energies of isoxanthohumol to a given protein.
** Reported experimental connection with anticarcinogenic function
*** Abbreviations stand for: XH - xanthohumol, 6P – 6 – prenylnaringenin and 8P – 8 - prenylnaringenin

Table S2. Potential Human Protein Targets of 8-prenylnaringenin

PDB ID with Chain	Protein Name	Predicted Docking Score [arb. units] *	Protein function	Anticarcino genic Function**	Other Docked Ligands*** (docking score)
3eigA	Dihydrofolate reductase	-59.89	Converts dihydrofolate into tetrahydrofolate and plays a crucial role in the cell metabolism and cellular growth. [7]	Yes [8]	IXH (-55.73)
4kmy A	Folate receptor beta	-57.38	Folate receptor beta a one member of the folate receptor family. Folate receptor beta is in conjunction with folate receptor alfa attached to cell membrane by glycosylphosphatidylinositol anchors. Both receptors are overexpressed in tumor cells and in tumor-associated macrophages. [3]	Yes [3]	IXH (-57.21)

4b6lA	Serine/threonine -protein kinase PLK3	-56.74	Polo-like kinases (PLKs) are the family of proteins that regulate DNA replication, mitosis and stress response. In cancer cells, these kinases are usually dysregulated and promote the uncontrolled cell proliferation and aberrant cell division. [15]	Yes [15]	-
1he8A	Phosphatidylinositol 3-kinase catalytic subunit, gamma isoform	-56.38	Phosphatidylinositol 3-kinase is a family of dimeric cellular components, which consist of a regulatory and a catalytic subunit. Both forms of phosphatidylinositol 3-kinase play an important role in lipid and protein kinase activity. Phosphatidylinositol 3-kinase also regulate diverse cellular functions such as proliferation, survival metabolism and motility. [16]	Yes [16]	-

* Knowledge-based docking scores with arbitrary units and represent relative binding free energies of a 8 - prenylnaringenin to a given protein.
** Reported experimental connection with anticarcinogenic function
*** Abbreviations stand for: **XH** - xanthohumol, **IXH** – isoxanthohumol and **6P** – 6 - prenylnaringenin

Table S3. Potential Human Protein Targets of 6-prenylnaringenin.

PDB ID with Chain	Protein Name	Predicted Docking Score [arb. units] *	Protein function	Anticarcinogenic Function**	Other Docked Ligands*** (docking score)
3zmv A	Lysine-specific histone demethylase 1A	-66.27	Lysine-specific histone demethylase 1A is an enzyme which modifies histones. Its main function is to regulate the expression of many genes, which are important for cancer progression and proliferation. [17]	Yes [17]	-
4b5p A	α-tubulin N-acetyltransferase	-62.83	Alpha-tubulin N-acetyltransferase is a regulator of α-tubulin acetylation. By the cause of regulation of Wnt1 and its downstream genes expression, the α-tubulin N-acetyltransferase is required for colon cancer proliferation and invasion. [18]	Yes [18]	-

3s9nC	Transferrin receptor protein 1	-60.80	The transferrin receptors act as the most important controls, that are mediated by receptors. The transferrin receptor 1 and transferrin receptor 2 are subtypes of the transferrin receptor family. The transferrin receptor 1 is abnormally expressed in cancer cells. One finds studies, that clinical drugs and antibodies targeting transferrin receptor 1 have showed strong anti-tumor effects. [19]	Yes [19]	-
1qibA	72 kDa type IV collagenase	-60.12	The activity of 72kDa type IV collagenase may occur on many levels, such as transcriptional mechanisms, of extracellular activation of latent enzyme. The invasion of human tumor cells through basement membranes may be the result of the net type IV collagenolytic activity. [9]	Yes [9]	IXH (-55.59)
2rgcA	GTPase HRas	-59.45	GTPase HRas is a member of a considerable family of enzymes, whose function is to bind and split GTP. GTPase HRas is involved in cell-to-cell communication, protein translation (in ribosomes) and in process of apoptosis. The GTPase HRas protein is recognised in 30% of human tumors. [20]	Yes [20]	-
4ywy A	Glutaminyl-peptide cyclotransferase	-59.34	Glutaminyl-peptide cyclotransferase is an enzyme, whose main function is to catalyze the posttranslational modification of an N-terminal glutamate of proteins to pyroglutamate. [21]	No	-
1w6jA	Lanosterol synthase	-59.02	Lanosterol synthase is the most important enzyme in the biosynthesis pathway of cholesterol. [22]	No	-

3gftA	GTPase KRas	-58.94	GTPase KRas is a member of aconsiderable family of enzymes, that bind and split GTP. The P53 tumor suppressor is supressed by the GTPase KRas due to activating the NRF2-regulated antioxidant system in cancer cells. [23]	Yes [23]	-
2jgbA	Eukaryotic translation initiation factor 4E type 2	-58.94	Overexpression of proteins included in the cell growth pathway, angiogenesis and survival factors, forced the eukaryotic translation initiation factor 4E type 2 to be overexpressed in fibroblasts or epithelial cells, which leads to the induction of cellular transformation and tumorigenesis. [24]	Yes [24]	-
5synA	Acyl-protein thioesterase 2	-58.59	Involved in depalmitoylation. [1]	Yes [1]	XH (-64.13) IXH (-65.36)
3k22A	Glucocorticoid receptor	-58.44	Glucocorticoid receptor mediates the effects of glucocorticoids (steroid hormones) by the intracellular ligand activating transcription factors. The glucocorticoid receptor is recognised as a novel mediator of proliferation in metastatic colorectal cancer cells. [25,26]	Yes [26]	-
2zjjA	Beta-secretase 1	-58.40	Beta-secretase 1 is an enzyme, which is involved in the proteolytic cleavage of the amyloid precursor protein. The recent studies revealed the differential expression of beta-secretase 1 in breast cancer tissues. [27]	Yes [27]	-

* Knowledge-based docking scores with arbitrary units and represent relative binding free energies of 6-prenylnaringenin to a given protein.
** Reported experimental connection with anticarcinogenic function
*** Abbreviations stand for: **XH** - xanthohumol, **IXH** – isoxanthohumol and **8P** - 8-prenylnaringenin
